# The Impact of Antipsychotic Polytherapy Costs in the Public Health Care in Sao Paulo, Brazil

**DOI:** 10.1371/journal.pone.0124791

**Published:** 2015-04-08

**Authors:** Denise Razzouk, Monica Kayo, Aglaé Sousa, Guilherme Gregorio, Hugo Cogo-Moreira, Andrea Alves Cardoso, Jair de Jesus Mari

**Affiliations:** 1 Centro de Economia em Saúde Mental (CESM), Department of Psychiatry, Universidade Federal de Sao Paulo, Sao Paulo, Sao Paulo, Brazil; 2 Department of Psychiatry, Universidade Federal de Sao Paulo, Sao Paulo, Sao Paulo, Brazil; Institute of Psychiatry, UNITED KINGDOM

## Abstract

**Introduction:**

Guidelines for the treatment of psychoses recommend antipsychotic monotherapy. However, the rate of antipsychotic polytherapy has increased over the last decade, reaching up to 60% in some settings. Studies evaluating the costs and impact of antipsychotic polytherapy in the health system are scarce.

**Objective:**

To estimate the costs of antipsychotic polytherapy and its impact on public health costs in a sample of subjects with psychotic disorders living in residential facilities in the city of Sao Paulo, Brazil.

**Method:**

A cross-sectional study that used a bottom-up approach for collecting costs data in a public health provider´s perspective. Subjects with psychosis living in 20 fully-staffed residential facilities in the city of Sao Paulo were assessed for clinical and psychosocial profile, severity of symptoms, quality of life, use of health services and pharmacological treatment. The impact of polytherapy on total direct costs was evaluated.

**Results:**

147 subjects were included, 134 used antipsychotics regularly and 38% were in use of antipsychotic polytherapy. There were no significant differences in clinical and psychosocial characteristics between polytherapy and monotherapy groups. Four variables explained 30% of direct costs: the number of antipsychotics, location of the residential facility, time living in the facility and use of olanzapine. The costs of antipsychotics corresponded to 94.4% of the total psychotropic costs and to 49.5% of all health services use when excluding accommodation costs. Olanzapine costs corresponded to 51% of all psychotropic costs.

**Conclusion:**

Antipsychotic polytherapy is a huge economic burden to public health service, despite the lack of evidence supporting this practice. Great variations on antipsychotic costs explicit the need of establishing protocols for rational antipsychotic prescriptions and consequently optimising resource allocation. Cost-effectiveness studies are necessary to estimate the best value for money among antipsychotics, especially in low and middle income countries.

## Introduction

In the last decade, antipsychotic polytherapy has been subject of controversy and criticism in the treatment of schizophrenia and related psychoses [1]. Different from psychotropic polytherapy (i.e., a combination of psychotropic drugs from different pharmacological classes), which may be indicated for the management of comorbidities in any psychiatric illness or in the treatment of mood disorders [2,3], the antipsychotic polytherapy (or polypharmacy) may be defined as a concurrent use of more than one antipsychotic with the purpose of a better control of psychotic symptoms [4,5]. Basically all schizophrenia treatment guidelines recommend the use of antipsychotic monotherapy and, as a second step, if there is no adequate response, switching to a second antipsychotic in monotherapy. In case of two treatment failures, or in some cases, three treatment failures, the patient is considered treatment-resistant, and the recommendation is the use of clozapine, the only approved antipsychotic for treatment-resistant schizophrenia [6–9]. Antipsychotic polytherapy is usually recommended by the algorithms as a last resource, as an attempt to augment clozapine effect [6–10], but even the combination with clozapine has a weak evidence of efficacy [11].

In terms of efficacy, there is no antipsychotic, whether typical or atypical, with a clear superiority over the others, with the exception of clozapine, which is more efficacious in cases of treatment-resistant schizophrenia [12,13]. Antipsychotics differ substantially in side-effects and currently they may be all equally considered first line treatment for psychosis, when only clinical response is taken into account, as we observe in the treatment guidelines [6–8].

Given the fact that, with the exception of clozapine, all antipsychotics do not differ substantially in terms of efficacy and virtually all guidelines recommend antipsychotic monotherapy for the treatment of psychoses, it is noteworthy that the use of antipsychotic polytherapy has increased [14–17].

According to a study based on chart review, most polypharmacy prescriptions have justifiable clinical rationales, such as refractoriness, problems of adherence, poor control of symptoms and use of a second antipsychotic for other reasons [1]. In other cases, polypharmacy could reflect an attempt of augmentation therapy, or a treatment of different symptoms domains (e.g., cognitive or negative symptoms) or even prescribing habits [5]. However, there is some evidence that it is reasonable to switch to monotherapy patients receiving polypharmacy, with benefits such as weight loss [18] and decrease of side effects, risk of drug interactions and costs [10].

Whether clinically justifiable or not, antipsychotic polypharmacy has not proven superior efficacy over monotherapy, and because it is so widely used, it is important to evaluate the economic impact of this practice.

The costs and economic impact of antipsychotic polypharmacy are relevant for the budget in mental health services [14]. Antipsychotic polytherapy represents a common prescribing pattern for severely ill patients living in residential facilities [19], but it is associated with higher health services costs and higher utilization of health services[20].

In the State of Sao Paulo, Brazil, six atypical and eight typical antipsychotics (including two depot) are freely available in the public health system to all citizens. Despite some governmental initiatives to elaborate treatment guidelines, the antipsychotic costs were not considered in the recommendations and no treatment algorithm was effectively implemented in the mental health care system. There is a huge variation of costs among antipsychotics and a lack of information about the costs of different patterns of antipsychotic prescription. Accordingly, we aimed to estimate the costs of antipsychotic polypharmacy and its impact on public health care costs in a sample of patients with psychotic disorders living in residential facilities in the city of Sao Paulo.

## Methods

### Study Design

A cross-sectional study was carried out in the city of Sao Paulo, from February 2011 to May 2012. This study is part of a research project that evaluated the direct costs of health services for a sample of subjects discharged from long-term psychiatric hospitals.

Setting: Residential facilities were created in Brazil under public government funding in 2000, to support people discharged from long-term psychiatric hospitals. There are two types of services: Residential facilities type 1, where six to eight people with mental disorders can live independently, sharing a house in the community, with an off-site carer available in an on-call basis, and Residential services type 2, for people with lower level of autonomy, in need of permanent care and with no family relationships. Type 2 is a fully-staffed home, with up to eight residents living there under 24-hour supervision of two carers taking turns working in 12 hours daily-basis regime. Carers should support all residents' needs and also help on the psychosocial rehabilitation process, in order to improve their autonomy and social skills. These services are linked to a Center of Psychosocial Rehabilitation (CAPS), a mental health center with specialists on clinical and psychosocial treatment for moderate and severe psychiatric disorders. Carers are also responsible to dispense all the medicines prescribed by psychiatrists in CAPS. They record all the activities related to the residents, in files kept in the residential facilities type 2. Until 2010, approximately 9,000 people were living in psychiatric hospitals in Brazil [21], and of this total, 6,349 were in the State of São Paulo [22]. In 2008, there were 160 people living in psychiatric hospitals in the city of São Paulo (capital of the State of São Paulo), and there was only one type 2 residential service and no type 1 residential facility. In 2011, at the beginning of this study, 19 fully-staffed residential facilities (type 2) had been created in the city of São Paulo, with 8 residents each.

Sampling: In 2008, two psychiatric hospitals were closed, and all the 160 inpatients were transferred to the 20 existing residential facilities in the city of São Paulo or to a psychiatric hospital in another city. No hospital records of these patients were available for data collection. At the beginning of this study sampling, in 2010, there were 151 people discharged from the two hospitals living in the residential facilities, and they were all selected to participate, according to the following inclusion criteria: being a former resident of a psychiatric hospital for one year or more and being able to understand and answer to the questions in the interviews.

### Ethics

This study was approved by the Ethical Committee of the Secretary of Health of the city of Sao Paulo. Carers and residents were adequately informed of all aspects regarding the participation and the purpose of the study, providing a written consent prior to the interviews.

### Assessments Tools

Seven trained researchers interviewed the carers and residents. Instruments used to assess demographic and clinical characteristics of the sample were: the Mini International Neuropsychiatric Interview (MINI) [23] for psychiatric diagnosis, the Clinical Global Impression- Severity subscale (CGI-S), to assess symptoms severity [24], the Quality of Life Scale [25], the Social Behavior scale [26], the Independent Living Skills Scale[27]. All the assessment tools were translated and adapted to Portuguese.

#### Mini International Neuropsychiatric Interview (MINI)

A validated Brazilian version 5.0 of Mini International Neuropsychiatric Interview [28] was used to assess lifetime and current psychiatric diagnosis according to DSM-IV and ICD-10. This is a short semi-structured diagnostic psychiatric interview developed to be used in clinical settings and for research [23].

#### Clinical Global Impression (CGI)

In order to assess the severity of psychiatric symptoms, a validated Brazilian version of Clinical Global Impression—Severity (CGI-S) [29] was applied. The score of the CGI-S based on behavior, global functioning and symptoms in the last seven days. This scale is composed of five dimensions (positive, negative, cognitive, depressive symptoms, and global). Scores range from 1 (normal) to 7 (the highest level of severity) [24].

#### Quality of life

The Brazilian version of the Quality of life Scale [30], was used to assess the resident quality of life in the last three weeks. This scale was developed for people with severe mental disorders [25].

#### Functioning

The Independent Living Skills Survey [27], Brazilian version [31], was used to assess functional living skills in the following domains: food, health, transport, self-care, money management, work and global autonomy. It assesses the frequency of some behaviors from “never occurs” (score 0) to “always occurs” (score 4) during the last month. Higher scores suggest better functioning. This instrument was collected from the resident carer.

The Social Behavior Scale (SBS) [26], Brazilian version [32], was used to assess social functioning. It includes 21 items which cover the daily social behavior in the last month (last 30 days), with information provided by the carer. Each item is scored from 0 (normal) to 4 (severe behavior problem). Responses were categorised into a binary variable: normal social functioning (score of 0 or 1) and impaired social functioning (score of 2 or higher).

#### Costs

The Client Sociodemographic and Service Receipt Inventory (CSSRI) [33] is a semi-structured instrument used to assess social and demographic data, accommodation data, detailed information about treatment, professional visits, and social and health services utilization. The CSSRI was translated to Portuguese and adapted to Brazilian context by Sousa et al (*Inventário Sociodemográfico de Utilização e Custos de Serviços*—ISDUCS) [34]. The CSSRI uses the patient and caregiver as information source.

### Data Collection

Data were collected by semi-structured and structured interviews conducted by trained psychiatrists, directly with residents and carers; some additional information could be obtained from the records available in the residential services. The SBS and the ILSS were applied with the carers, as such scales rely on the information obtained from the carers' through the observation of the residents' behavior.

The QLS, the MINI and the CGI-S were applied by psychiatrists directly with the residents. The CSSRI was used to assess sociodemographic characteristics, previous and current occupational status, pattern of service uses, medicines and treatment received. Input to CSSRI were obtained from the interviews with both carers and residents, as well as from the records.

### Definition of antipsychotic polytherapy

In this study we considered as antipsychotic polytherapy only those residents who were regularly taking two or more antipsychotics (on a daily basis for oral and weekly/monthly in case of depot) in the last 30 days previous to the interview. Data related to occasional antipsychotic use were also collected, but was not considered polytherapy. Antipsychotic monotherapy was defined as the use of only one antipsychotic, even when other non-antipsychotic drugs were taken concomitantly. The combination of one antipsychotic with other drug classes (e.g. benzodiazepines or antidepressants) was not considered an antipsychotic polytherapy in this study.

Carers were responsible to dispense and supervise all the medicines taken by the residents, and all the information was daily recorded. The antipsychotics were freely provided by the public health system. The typical antipsychotics included haloperidol, chlorpromazine, levomepromazine, periciazine, thioridazine, pimozide, pipotiazine depot and haloperidol depot. Atypical antipsychotics included risperidone, quetiapine, olanzapine, ziprasidone, aripiprazole and clozapine.

### Estimation of direct costs

A bottom-up approach was used for the estimation of direct costs, according to the public health service provider perspective.

The CSSRI allows calculating health care costs using the modality of service or intervention and the frequency of use. Direct costs of residential care, health care and treatments were calculated for each resident, for the year 2011.

Direct costs of the public health services for a 30-day period included: inpatient costs + outpatient costs + emergency costs + medicine costs + psychosocial interventions (e.g. psychotherapy, occupational therapy) costs + transport costs (ambulance).

### Estimation of unit of costs

All costs were estimated for the year 2011 and the estimation of unit cost for medicines was based on Brazilian Government Drug Price Database [35]. This database is available online for public consultation and contains information about the costs of medicines per unit, as paid by the government, according to the city, year, posology, pharmaceutical companies and institutions. On average, the public health services buy medicines with prices up to 30% lower than the market prices. The estimation of costs of antipsychotic polytherapy was calculated by summing up all costs of the antipsychotics regularly used in a 30-day period. The month of July, 2011 was used as reference for all the costs.

The estimation of cost for accommodation services was obtained directly from the residential managers. Information about residential costs included: electricity, gas, water, rent, repairs, transport, human resources, food and house supplies and overhead. The estimation of costs for public health services included hospital, emergency, outpatient and community services and transport costs. The estimation of health services costs was based on the frequency of service use in the previous month and on the calculation of the unit of cost: per visit (consultations) and per day (hospitalization). Units of costs for health services were directly obtained from service managers.

### Statistical analyses

Two groups (antipsychotic polytherapy group and monotherapy group) were compared through non parametric test (Chi square or Fischer exact test for categorical and nominal variables; significance level p<0.05) in terms of social and demographic characteristics, severity of psychiatric symptoms, psychiatric diagnoses, medicines, drug association with benzodiazepines, and type of antipsychotic used.

A multivariate linear regression analysis was fitted with direct costs of public health care per person per month as dependent variable, and the number of antipsychotics, geographical location of the facility, olanzapine use, and the length of time living in the residential facility were included as covariates. Direct costs of public health care included costs with inpatient, outpatient, emergency and community health services and treatment received in the previous month (including all medicines).

Firstly, univariate linear regressions were built considering, individually, different covariates, exploring the predictive of each measure on the costs of the residential facilities; then a multivariate linear regression analysis was fitted only with those covariates statistically significant in the univariate analysis (adopting significance level at 0.05).

## Results

Four out of 151 residents refused to participate to this study. One hundred forty seven subjects were included in the sample; 134 used antipsychotics regularly; the rate of antipsychotic polytherapy (two or more antipsychotics in daily use) was 38% (N = 56) (Table 1). Demographic and clinical characteristics of the sample are also described in Table 1. There were no differences between those in use of antipsychotic polytherapy and monotherapy regarding sex, age, education, severity of psychiatric symptoms, cognitive or negative symptoms, psychiatric diagnosis, pharmacological treatment for clinical somatic symptoms (hypertension, diabetes, hypercholesterolemia) and benzodiazepine use. Antipsychotic polytherapy was correlated with regular use of haloperidol, chlorpromazine, levomepromazine and depot antipsychotic but it was not correlated with regular use of any other antipsychotic.

**Table 1:** Demographic and clinical characteristics according to drug regimen (antipsychotic polytherapy or monotherapy) (N = 134)*.

	Antipsychotic Polytherapy (more than 1 antipsychotic)**		p (<0.05)
	No	Yes	
**Gender**			NS
male	41	26	
female	37	30	
**Education**			NS
illiterate	15	09	
primary	54	42	
High school	08	02	
College	01	02	
**Severity of psychiatric symptoms (CGI scale)**			NS
Mild or absent (0–2)	29	19	
Moderate to severe (3–7)	49	37	
**Severity of cognitive symptoms (CGI scale)**			NS
Mild or absente (0–2)	32	24	
Moderate to severe (3–7)	45	32	
**Negative symptoms (CGI scale)**			NS
Mild or absente (0–2)	45	30	
Moderate to severe (3–7)	32	26	
**Psychiatric diagnosis**			NS
none	7	5	
Alcohol or drugs problems	2	5	
Psychosis	52	36	
Psychosis and drug or alcohol problems	05	05	
Psychotic depression	1	3	
Depression	2	0	
Bipolar mania	5	1	
missing	4	1	
**Benzodiazepine use (previous month)**			NS
no	47	35	
yes	31	21	
**Depot antipsychotic use (previous month)**			**p<0.01**
no	51	12	
yes	27	44	
**Haloperidol use (previous month)**			**p<0.01**
no	51	12	
yes	27	44	
**Chlorpromazine use (previous month)**			**p<0.01**
no	66	23	
yes	12	33	
**Medicine use for metabolic syndrome (hypertension, diabetes, cholesterol)**			NS
no	44	40	
yes	34	16	

*Daily use of only one antipsychotic regardless of the association with drugs from other pharmacological classes,

**daily use of 2 or more antipsychotics

Overall, the mean (SD) length of time living in residential service was 35 (15.4) months. The mean length of time living in residential service was significantly lower in polypharmacy group than in monotherapy group (p = 0.011). The mean (SD) length of time living in psychiatric hospitals were 115.39 (106.02), with no significant difference among them.

The mean (SD) number of individual psychotropics use was 3.37 (1.4), range 0 to 7, and the mean (SD) number of individual antipsychotics was 1.45 (0.9), range 0 to 5.

Patterns of antipsychotic prescriptions were: 45.5% (n = 67) used at least one atypical antipsychotic, 64.6% (n = 95) used at least one typical antipsychotic, 14.2% (n = 21) used one typical and one atypical antipsychotic, 13.6% (n = 20) used two typical antipsychotics, and 10.8% (n = 16) used three or more antipsychotics. The use of clozapine use was below 5% (Table 2).

**Table 2:** Frequency, mean dose and unit of costs of most prescribed psychotropic (n = 147).

	n (%)	mean ±SD (mg/day)	min/max (mg/day)	Pill unit mg	Unit cost (per pill) R$	Total sum cost per day R$
**Chlorpromazine**	45 (30.6)	250.6 ± 175.7	100–800	100	0.05	6.13
**Haloperidol**	78 (48.3)	4.0 ±5.7	1.0–25.0	5	0.01	1.57
**Periciazine**	11 (7.5)	25.7±20.0	5.0–60.0	1	0.01	3.84
**Levomeprazine**	06(4.1)	175.0±108.4	50–300	100	0.26	2.75
**Thioridazine**	01(0.68)	-	300	100	0.18	0.06
**Risperidone**	21 (14.2)	4.3± 2.2	1.0–8.0	2	0.05	3.18
Olanzapine*	18 (12.2)	16.1±5.8	10–30	10	20.00	527.05
**Clozapine**	07 (4.8)	335.7±188.7	150–700	100	2.00	57.00
**Quetiapine**	09 (6.1)	455±113	300–600	200	7.33	224.45
**Ziprasidone**	07 (4.8)	160±65.3	80–240	80	7.20	103.16
**Aripiprazole**	02 (1.4)	-	30	15	14.96	59.79
Haloperidol decanoate**	13 (8.8)	140.9±78.1	50–300**	50	0.04	1.54
Pipotiazine depot**	02 (1.4)	-	50–75**	25	0.39	1.93
**Total costs antipsychotics**						989.44
**Carbamazepine**	30 (26.5)	663±294.5	200–1600	200	0.05	6.16
**Valproic acid**	22 (14.9)	1318±546.5	500–3000	500	0.04	2.26
**Lithium**	09(6.1)	857.1±320.7	300–1200	300	0.06	14.44
**Biperiden**	59 (40.0)	3.4±1.6	2–8	2	0.04	0.038
**Prometazine**	41 (27.9)	48.1±20.0	25–100	25	0.02	1.62

* Two values were used to olanzapine unit costs (R$10.00 and R$20.00).

** Dose per month

Antipsychotics agents were also frequently associated with benzodiazepines, carbamazepine, valproic acid, biperiden and promethazine (Table 2). The use of three or more psychotropics (including pharmacological classes other than antipsychotics) was present in 75% of the sample.

The total direct costs of public health care (health care, medicines and accommodation) for a 30-day period were R$ 529,425.26. The total costs of accommodation were R$ 470,213.78, the total costs of health care were R$ 24,058.45 and the costs of medicines were R$35,153.03. The total costs of psychotropic were R$31,658.29. The costs of antipsychotics corresponded to 94.4% of the total psychotropic costs and to 49.5% of all health services use when excluding accommodation costs of the residential facilities.

The mean monthly cost of antipsychotic polypharmacy per person varied according to the type of antipsychotics combination: typical- atypical costs were R$515.64 (R$457.1), while two-typical antipsychotics combination costs were R$8.72 (R$8.1). Olanzapine costs (n = 18) corresponded to 51% of all psychotropic costs and 53% of the total antipsychotic costs.

A regression analysis was performed to explore a model in which direct public health care costs (sum of health services, medicines and accommodation costs for a 30-day period) was the dependent variable.

Table 3 shows the estimates (in R$) for different covariates on the costs of the residential facilities individually; hence, for each used covariate, a different linear regression was built. Four covariates were statistically significant: the number of antipsychotics (for each antipsychotic medicine, there is an increase in the residential facilities costs by R$175.14), the geographical location of the facility (taking North zone as reference, the South region was more expensive by R$484.29), and olanzapine use (taking other antipsychotic drugs as reference, olanzapine was more expensive by R$1,014.91), and the length of time living in the residential facility (there is a decrease of R$8.5 per month).

**Table 3:** Variables explaining health care costs (including residential facilities costs).

	Estimate (R$)	Standard Error	p-value	95% CI		r^2^
**Age**	-5.715145	4.598371	0.216	-14.8036	3.373349	0.0105
**Region South**	484.2919	212.4812	**0.024**	64.25678	904.327	0.1099
**Region East**	-270.1016	181.2046	0.138	-628.309	88.10557	
**Region Southeast**	160.6955	161.2279	0.321	-158.022	479.4127	
**Region Center-west**	-277.9313	166.447	0.097	-606.966	51.10303	
**Lenght of time living in residential facility (months)**	-8.56085	3.877724	**0.029**	-16.225	-0.89669	0.0325
**Olanzapine regular use (reference: other antypsychotics)**	1014.918	165.3593	**<0.001**	688.0728	1341.763	0.2191
Number of antipsychotics*	175.1402	66.6714	**0.01**	43.36682	306.9135	0.0454
**CGI score**	8.826827	42.42076	0.835	-75.0161	92.66974	0.0003
**SBS score**	-0.0748829	5.204153	0.989	-10.3607	10.21091	<0.00001
**ILSS**	-3.294516	9.786797	0.737	-22.6377	16.04869	0.0008
**Number of psychotropics**	76.48758	43.18387	0.079	-8.8636	161.8388	0.0212
**Lenght of time living in psychiatric hospital**	0.9498517	0.53065	0.076	-0.09896	1.99866	0.0216

CGI: Clinical Global Impression; ILSS: Independent Living Skills Survey; SBS: Social Behavior Scale.

*Number of antipsychotics drugs refers to the number of antipsychotics regularly used (in this sample residents used 1 to 5 antipsychotics).

The outcome (direct costs of public health care) and its residual distributions were non-normal, as usually cost data are. However, even under non-normal error distribution linear regression and its estimates are BLUE (best linear unbiased estimator), being robust in a technical sense; in other words, it is computable under almost any circumstance.

In Table 4, only the statistical significant isolated covariates were used to compose a multivariate linear regression with the four above cited covariates. The variance explained by the four covariates together is 30.9%, remaining statistically significant the location in South region and the regular use of olanzapine.

**Table 4:** Multivariate linear regression of the the variables with most impact in residential costs.

Variables	Estimate (R$)	Standard Error	p-value	95% CI	
**Location of the facility**					
*South*	520.9643	192.3548	**0.008**	140.6205	901.308
*East*	-95.0554	167.5138	0.571	-426.281	236.1703
*Southeast*	119.185	151.3616	0.432	-180.103	418.4728
*Center-west*	-11.9501	169.8589	0.944	-347.813	323.9125
**Olanzapine regular use**	945.8309	163.1091	**<0.001**	623.3146	1268.347
**Number of antipsychotics**	105.032	72.91305	0.152	-39.1393	249.2032
**Length of time living in the facility (months)**	-4.05731	3.837409	0.292	-11.645	3.530418

Table 5 shows the same model run under bootstrapping standard errors and normal based 95% confidence interval. Bootstrapping is a nonparametric statistical technique and it was used because it does not require distribution assumption providing accurate inferences when the data are not normally distributed.

**Table 5:** Multivariate linear regression of the the variables with most impact in residential costs (Bootstrapping).

Costs variables	Observed coefficient (R$)	Bootstrap Standard error	p-Value	95% CI
**Residence location**				
*South*	440.4302	219.5569	0.045	10.10654–870.7538
*East*	-201.0942	173.0284	0.245	-540.2237–138.0352
*Southeast*	38.98181	234.3863	0.868	-420.4069–498.3705
*Center-west*	-179.2897	136.9568	0,191	-447.7201–89.14073
**Olanzapine regular use**	269.1076	95.10215	0.005	82.71086–455.5044
**Number of antipsychotics**	206.4687	108.099	0.056	-5.401545–418.3389
**Length of time living in the facility (months)**	-2.673395	2.788026	0.338	-8.137825–2.791035

## Discussion

The rate of antipsychotic polypharmacy observed in our sample (38%) is within the range observed in other studies [10,19,34,35]. The practice of polypharmacy was not correlated with the severity of psychiatric symptoms, and in many cases would not be clinically justified. Given the fact that approximately 30% of patients with psychosis are treatment-resistant [36], we can assume that the prescription of clozapine in our sample was very low (<5%), opposed to all guidelines recommendations. It is possible that some cases of switches and cross-titration have been included, but the percentage might be minimal, as the data were based in the previous month, and the patients were in chronic states, living in residential facilities for a mean period of 3 years.

Studies estimating the costs of antipsychotic polypharmacy are scarce. Instead, most studies estimated the costs and cost-effectiveness of a specific monotherapy treatment. In this regard, there is no evidence on the clinical superiority and on cost-effectiveness among antipsychotics, with the exception for clozapine for severe psychotic disorders [37].

The choice of an antipsychotic therapy is strongly associated with health services costs. Atypical antipsychotic monotherapy costs can be 167 times greater than typical antipsychotic monotherapy costs [38]. A Brazilian cost-utility study reported that the economic impact of olanzapine on public health services was greater than risperidone and haloperidol [39]. In our study, almost half of direct health care costs (excluding the residential facilities) were due to antipsychotic costs, with olanzapine costs corresponding to more than half of all psychotropic costs. The unit cost of olanzapine was 400 times greater than the unit cost of risperidone in our study. Typical antipsychotics have been recommended for low and midlle income countries because of the high costs of the atypicals [40], but it is not the case in Brazil. However, it is important to take attention from policymakers about the consequences on buying new expensive drugs without taking into account clinical effects and costs. Olanzapine in this case is only an example, demonstrating the need for more cost-effectiveness studies exploring the range of costs (threshold) that would be suitable for a country to afford and have relevant benefits. Our study does not directly address cost-effectiveness but it is the first study in Brazil that measure the effect of drug costs on services and to some extent, this can be an alert to discuss the rational use of antipsychotic in different settings, considering low and middle income countries.

The lower rate of clozapine use is not justified by its cost; assuming the unit costs used in this study, the cost of clozapine therapy (450mg/daily) plus four complete blood counts (CBC) per month would be 50% of the costs of olanzapine therapy with a daily dose of 10 mg. Therefore, protocols for rational prescription of antipsychotics might be crucial for optimizing budget allocation. The reason for this low clozapine use should be better investigated, but one hypothesis is the difficulty to guarantee the weekly blood counts needed with this drug, or maybe the lack of training on its use. In Brazil, the choice of medicines is usually based on the physician’s preferences and the majority of professionals don’t take the costs and price into account.

A North-American study demonstrated that the combination of two antipsychotics added annually U$4,244.00 per person when compared with monotherapy [40]. In our study, an antipsychotic polytherapy regimen added annually R$ 2,755.00 (approximately U$1,375.00) per person for each antipsychotic added to the monotherapy.

The present study has some limitations. This was a cross-sectional study and data was collected retrospectively, considering only the month previous to the interview; as a result, variations in the treatment over the year could not be accurately captured. Although the sample size may be considered small, it is not a comparative study, and almost all residents discharged from two psychiatric hospitals in the city of Sao Paulo were included. It was not the aim of the study to compare the rate of antipsychotic polytherapy in the residential facilities with the rate found in other settings, such as hospitals and outpatients services.

The unit of costs for psychotropic was based on Federal Government database [35], and it may not represent the real costs at a regional level. Although we simulated two values of unit costs for olanzapine (R$ 10.00 and R$20.00) and results remained similar, sensitivity analyses were not carried out to verify potential variation on other drug costs. This was not a cost-effectiveness study, and decision of budged allocation should not be based only on costs. Moreover, the harms of antipsychotic polytherapy were not considered, such as higher incidence of possible side-effects, such as weight gain and complications of drug interactions.

## Conclusions

In summary, antipsychotic polytherapy adds a huge economic burden to health care systems, and the choice of the antipsychotic may negatively impact the financial health care management. The routine prescription of antipsychotics should be evaluated in terms of clinical benefits, cost-effectiveness, potential harms and financial burden to health care. More research is needed to explore how to monitor the rates of polytherapy, the costs and clinical consequences, and also to establish clear criteria for the rational use of antipsychotic polytherapy. Such practices could contribute to a better budget planning in mental health care management and, eventually, to better clinical outcomes. Considering the limited budget in low and middle income countries like Brazil, this study raises the attention to the adoption of better strategies on resource allocation, such as establishing a threshold for costs according to the benefits of newer drugs, through cost-effectiveness comparisons.
